# Catalytic Oxidative Removal of Volatile Organic Compounds (VOCs) by Perovskite Catalysts: A Review

**DOI:** 10.3390/nano15090685

**Published:** 2025-04-30

**Authors:** Tong Xu, Chenlong Wang, Yanfei Lv, Bin Zhu, Xiaomin Zhang

**Affiliations:** 1 Laboratory of Plasma Catalysis, Dalian Maritime University, Dalian 116026, China; heyangxv@dlmu.edu.cn (T.X.); chenlongwang@dlmu.edu.cn (C.W.); yanfeilv@dlmu.edu.cn (Y.L.); 2 State Key Laboratory of Catalysis, Dalian National Laboratory for Clean Energy, Dalian Institute of Chemical Physics, Chinese Academy of Sciences, Dalian 116023, China

**Keywords:** VOC removal, perovskite catalysts, catalytic oxidation, plasma catalysis

## Abstract

Volatile organic compound (VOC) emissions have become a critical environmental concern due to their contributions to photochemical smog formation, secondary organic aerosol generation, and adverse human health impacts in the context of accelerated industrialization and urbanization. Catalytic oxidation over perovskite-type catalysts is an attractive technological approach for efficient VOC abatement. This review systematically evaluates the advancements in perovskite-based catalysts for VOC oxidation, focusing on their crystal structure–activity relationships, electronic properties, synthetic methodologies, and nanostructure engineering. Emphasis is placed on metal ion doping strategies and supported catalyst configurations, which have been demonstrated to optimize catalytic performance through synergistic effects. The applications of perovskite catalysts in diverse oxidation systems, including photocatalysis, thermal catalysis, electrocatalysis, and plasma-assisted catalysis, are comprehensively discussed with critical analysis of their respective advantages and limitations. It summarizes the existing challenges, such as catalyst deactivation caused by carbon deposition, sulfur/chlorine poisoning, and thermal sintering, as well as issues like low energy utilization efficiency and the generation of secondary pollutants. By consolidating current knowledge and highlighting future research directions, this review provides a solid foundation for the rational design of next-generation perovskite catalysts for sustainable VOC management.

## 1. Introduction

With the rapid development of industry and the acceleration of the urbanization process, the emission problem of volatile organic compounds (VOCs) has become increasingly prominent and has become one of the environmental focuses attracting global attention [1,2]. VOCs pose great harm to both the environment and human health. In terms of the environment, VOCs are important precursors for the formation of photochemical smog and secondary organic aerosols, which can lead to a decrease in atmospheric visibility, trigger adverse weather phenomena such as haze, and severely damage the ecological balance [3,4,5]. As for human health, long-term exposure to an environment containing VOCs can irritate the eyes, respiratory tract, etc., causing symptoms such as coughing, asthma, and dizziness [6,7,8]. Moreover, some VOCs are carcinogenic, teratogenic, and mutagenic, seriously threatening human life and health [9,10]. According to statistics from the World Health Organization, the annual global VOC emissions from industrial sources exceed 150 million tons, and approximately 30% of respiratory diseases are directly related to VOC exposure [11]. In recent years, with the acceleration of the industrialization process and the continuous emergence of new pollutants, VOC pollution has shown a trend of complex sources and diverse components; its environmental fate and health risks have attracted high attention from society. VOCs and NO_x_ can generate ozone and secondary organic aerosols, which are the main causes of urban photochemical smog [12,13]. Some components, such as benzene series and polycyclic aromatic hydrocarbons, are classified as Group 1 carcinogens by the International Agency for Research on Cancer and can cause gene mutations and organ damage through respiratory exposure [14,15,16]. Currently, VOC emission sources cover three major categories: industrial processes (petrochemicals, coating), mobile sources (motor vehicle exhaust), and domestic sources (volatile substances from building materials) [17,18]. Among them, the proportion of benzene series emissions in the industrial coating process accounts for as high as 45%. It is worth noting that China’s 14th Five-Year Plan has included VOCs in the binding emission reduction indicators, requiring a reduction of more than 10% by 2025, which puts forward dual requirements for the improvement of treatment efficiency and the energy consumption optimization of VOC treatment technologies [19].

Currently, a variety of traditional technologies have been developed for the removal of VOCs, such as adsorption, combustion, and biological treatment methods. Among them, the adsorption method uses the adsorption of adsorbents to VOCs to achieve separation, but it has problems such as limited adsorption capacity and difficulty in adsorbent regeneration [20]. The combustion method oxidizes and decomposes VOCs through high temperature, but it has high energy consumption and is prone to secondary pollution [21,22]. Although the biological treatment method has advantages such as low cost and environmental friendliness, its treatment efficiency is greatly affected by the activity of microorganisms, and it has poor treatment effects on high-concentration and difficult-to-degrade VOCs [23]. The limitations of these traditional methods have prompted experts to continuously explore new technologies and methods. The catalytic oxidation technology for VOC removal has received extensive attention from scientists due to its great potential in solving existing technical problems. This technology reduces the activation energy of the reaction through the active sites on the surface of the catalyst and can achieve deep mineralization of VOCs at a relatively low temperature, and it also has the advantages of low energy consumption and no secondary pollution [24,25,26,27].

The core of the catalytic oxidation system lies in the development of high-performance catalysts [28,29]. Currently, catalysts used for VOC oxidation can be mainly divided into two categories: noble metal catalysts and non-noble metal catalysts [30]. Although noble metal catalysts (Pt, Pd-based) have excellent low-temperature activity, their scarcity and sintering deactivation problems lead to high costs [31,32]. Non-noble metal catalysts are mainly metal oxide catalysts (such as Al_2_O_3_, ZrO_2_, CeO_2_, SiO_2_, etc.). These catalysts do not contain noble metals; so, they are relatively inexpensive. However, due to their low specific surface area, they usually need to be operated at a relatively high reaction temperature to achieve efficient catalytic oxidation of VOCs, which limits their widespread application [33]. Against this background, perovskite-type catalysts (ABO_3_) stand out due to their unique crystal structure, tunable electronic properties, and excellent catalytic performance [34]. They not only have easily obtainable unique physical and chemical properties, such as dielectric, pyroelectric, magnetoresistive, and ferroelectric properties, but also facilitate the adjustment of structure and properties according to actual needs, such as constructing micro-nano pores, embedding nanoparticles, creating surface defects, modulating light response properties, etc. [35,36,37,38]. Therefore, they have become one of the research hotspots and have broad application prospects.

This review systematically combs through the recent research progress of perovskite catalysts in the field of catalytic oxidation of VOCs. It focuses on expounding the optimization effects of the relationship between crystal structure and activity, the electronic regulation mechanism, and the nano-engineering strategy on catalytic performance. By analyzing the synergistic effect of metal ion doping and carrier loading, the differentiated application mechanisms of perovskite materials in photocatalytic, thermocatalytic, electrocatalytic, and plasma synergistic catalytic systems are revealed. For the first time, the regulation laws of multi-dimensional modification strategies on the catalytic performance of perovskites are systematically integrated, and the catalyst design principles for complex industrial waste gases are summarized. Meanwhile, a trinity analysis framework of “structure-performance-application” is constructed. These research achievements not only provide theoretical support for the development of low-cost and highly stable perovskite catalysts, but also, by revealing the dynamic evolution mechanism of active sites, offer new ideas for breaking through the efficiency bottleneck of traditional catalytic systems.

## 2. Perovskite Catalysts

### 2.1. Crystal Structure and Characteristics

#### 2.1.1. Crystal Structure

**Figure 1:**
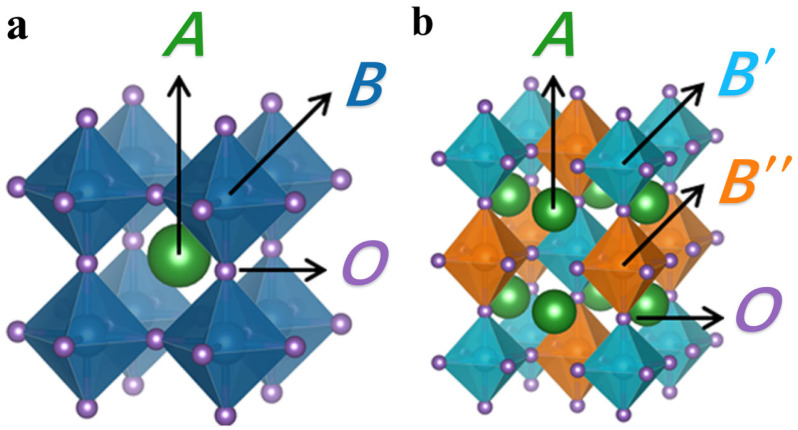
(**a**) Structure of perovskite ABO_3_, (**b**) structure of double perovskite A_2_B′B″O_6_ (Reproduced with permission from reference [39]).

Perovskite catalysts have a typical ABO_3_ structure; the crystal structure is shown in Figure 1a [39]. In the ideal perovskite structure, the A-site ions are usually rare earth metal ions with a relatively large radius (La^3+^, Ce^4+^, etc.) or alkaline earth metal ions (Ca^2+^, Sr^2+^, etc.), which are located at the center of the cuboctahedra voids formed by 12 oxygen atoms and form a 12-coordinate structure with the oxygen atoms. This relatively large ionic radius and the high coordination number enable the A-site ions to stably occupy the lattice positions, providing a basic framework support for the entire crystal structure [40,41,42]. The presence of A-site ions also has a certain influence on the electronic state and catalytic activity of B-site ions. By changing the type and doping ratio of A-site ions, the lattice parameters, oxygen vacancy concentration, and electronic structure of the catalyst can be adjusted, thereby affecting the catalytic performance of the catalyst [43,44].

The B-site ions are generally transition metal ions with a relatively small radius, such as Mn^3+^, Fe^3+^, Co^3+^, Ni^2+^, etc., which are located at the center of the octahedron composed of six oxygen atoms and form an octahedral structure with six coordination to the oxygen atoms [45]. The type and valence state of B-site ions play a crucial role in the catalytic activity of the catalyst. Since transition metal ions have abundant electron orbits and variable oxidation states, they can provide multiple active sites in the catalytic reaction and participate in the electron transfer and chemical reaction processes. Different transition metal ions have different electronic structures and redox abilities; so, they have different catalytic selectivities for different reactions [46,47]. For example, in the catalytic oxidation reaction of VOCs, Mn-based perovskite catalysts have high activity for the oxidation of toluene, while Co-based perovskite catalysts show better performance for the oxidation of formaldehyde [48].

In the ABO_3_ structure, the radius ratio of the A-site and B-site ions, as well as the valence states of the ions, needs to meet certain conditions to ensure the stability of the crystal structure. Goldschmidt proposed the concept of the tolerance factor (t) to describe the stability of the perovskite structure, as shown in Equation (1):

(1) $$t=\frac{\mathrm{rA}+\mathrm{rO}}{\surd 2(\mathrm{rB}+\mathrm{rO})}$$

Among them, rA, rB, and rO represent the radii of the A-site ions, B-site ions, and oxygen ions, respectively. When the value of t is between 0.8 and 1.0, the perovskite structure can exist stably; when t is close to 1, the crystal structure approaches the ideal cubic crystal system; when the value of t is greater than 1, the crystal structure will be distorted and non-cubic crystal system structures, such as the tetragonal crystal system, orthorhombic crystal system, or trigonal crystal system, may be formed [49]. The distortion of the crystal structure will lead to the generation of stress and defects in the lattice, affecting the electronic structure and oxygen vacancy concentration of the catalyst, thus having a significant impact on the catalytic performance. In some studies, it has been found that perovskite catalysts with an orthorhombic crystal system structure, due to their relatively large lattice distortion, generate more oxygen vacancies and active sites. It exhibits higher activity than catalysts with a cubic crystal system structure in the catalytic oxidation reaction of VOCs [50].

In addition, the crystal structure of perovskite catalysts also has a certain degree of flexibility. A-site and B-site ions can be partially substituted or doped with other elements to form a variety of derivative structures, such as double perovskite (A_2_B′B″O_6_) (Figure 1b) [39]. These derivative structures further enrich the structure and properties of perovskite catalysts by introducing different elements and regulating the atomic arrangement, providing more possibilities for their application in the field of catalysis. For example, the synergistic effect between different B-site ions in the double perovskite structure can significantly improve the catalytic activity and stability of the catalyst and exhibit excellent performance in some complex catalytic reactions.

#### 2.1.2. Electronic Properties

The unique crystal structure of perovskite catalysts endows them with special electronic properties which play a crucial role in their catalytic performance. In the ABO_3_ structure of perovskite, the electronic structures of A-site and B-site ions influence each other, jointly determining the energy band structure, electronic conduction characteristics, and redox properties of the catalyst [51].

In terms of the energy band structure, the valence band of perovskite catalysts is mainly composed of O 2p orbitals, while the conduction band is mainly composed of the d orbitals of B-site ions [52]. Due to the fact that the d orbitals of B-site transition metal ions have multiple energy levels, the energy band structure of perovskite is relatively complex, with multiple energy bands and band gaps. The size and distribution of these band gaps determine the catalyst’s light absorption ability and the recombination rate of electron-hole pairs. In photocatalytic reactions, a suitable energy band structure enables the catalyst to effectively absorb light energy, which excites the generation of electron-hole pairs, and thus promotes the progress of chemical reactions [53,54].

**Figure 2:**
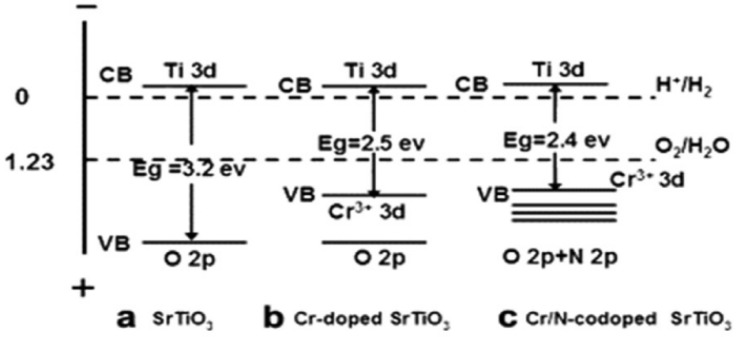
Electronic band structures of SrTiO_3_, Cr-doped SrTiO_3_, and Cr/N co-doped SrTiO_3_ (Reproduced with permission from reference [58]).

Meanwhile, the electron conduction properties of perovskite-type catalysts are also closely related to the electronic states of A-site and B-site ions. The presence of A-site ions can affect the electron cloud distribution of B-site ions [55,56]. Under the action of the crystal field, the d electrons of B-site transition metal ions will undergo energy level splitting, forming different electron orbitals. During the catalytic reaction, electrons can jump between these different electron orbitals, thus realizing electron conduction; this electron conduction property will directly affect the catalytic activity of the catalyst [57]. As shown in Figure 2, by doping elements with different electron-donating abilities at the B-site, the electron cloud density of B-site ions can be adjusted, and their redox potential can be changed, thereby improving the catalytic activity of the catalyst for specific reactions [58].

### 2.2. Catalytic Mechanism

The oxygen vacancies present in perovskite catalysts also have a significant impact on their electronic properties and catalytic performance. Oxygen vacancies refer to the vacancy defects formed due to the absence of oxygen atoms in the lattice. Due to the presence of oxygen vacancies, the electron cloud distribution around them will change, forming localized electronic states [59,60]. These localized electrons can participate in the catalytic reaction and act as active sites to promote the adsorption and activation of reactants. Meanwhile, oxygen vacancies can also regulate the electronic conduction performance of the catalyst [61]. By changing the migration path and concentration of electrons, they affect the catalytic rate and selectivity [62]. Theoretical calculation and analysis of the reaction kinetics model represent one of the most extensively employed approaches for researching and elucidating the catalytic reaction mechanism in VOCs. Regarding the oxidation of VOCs, there are essentially three main categories of kinetic models: (i) the power-law model; (ii) the surface reaction model, including the Langmuir–Hinshelwood model and the Eley–Rideal model; and (iii) the redox model, which is the Mars–van Krevelen model [63].

**Figure 3:**
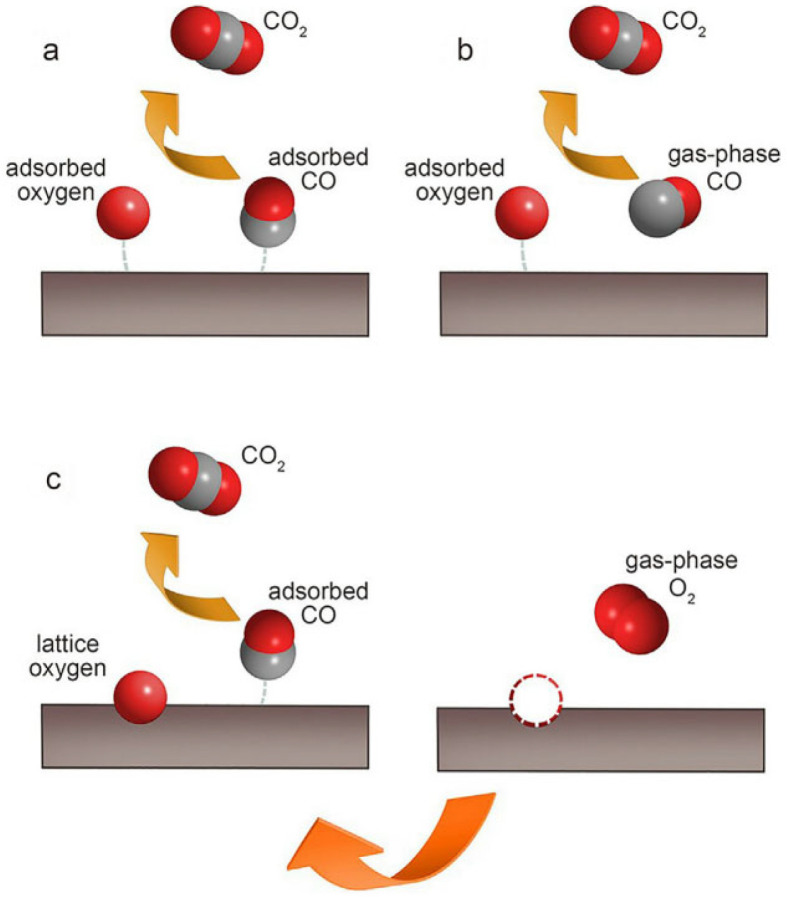
Three typical kinetic models of the CO oxidation process: (**a**) Langmuir–Hinshelwood model; (**b**) Eley–Rideal model; (**c**) Mars–van Krevelen model (Reproduced with permission from reference [63]).

The power-law model represents the most straightforward model, and it is applicable for elucidating the catalytic combustion process of pure VOCs, without considering the interference exerted by the reaction products. When the rate-limiting step happens during the processes of adsorption and surface reaction, the Langmuir–Hinshelwood model (the reaction taking place between the adsorbed oxygen species and the adsorbed VOC species) or the Eley–Rideal model (the reaction of adsorbed oxygen with gaseous volatile organic compounds) can be considered. In the Mars–van Krevelen model, initially, the catalyst undergoes reduction because of the reaction between the lattice oxygen and the adsorbed VOCs. Subsequently, it is oxidized by the oxygen present in the gas phase [64,65,66]. The oxidation of carbon monoxide over perovskite catalysts frequently serves as a probing reaction to elucidate the oxidation mechanism of other gaseous substances. Figure 3 shows the carbon monoxide oxidation processes in three typical kinetic models.

Studies have shown that in the catalytic oxidation reaction of VOCs, oxygen vacancies are one of the key factors, providing a site for the adsorption and activation of oxygen. Oxygen molecules are adsorbed at the oxygen vacancies and activated into active oxygen species such as O^2−^ and O^−^. These active oxygen species react with VOC molecules, which is equivalent to the participation of lattice oxygen in the process of oxidizing VOCs, causing the catalyst to be reduced. Subsequently, the oxygen in the gas phase oxidizes the reduced catalyst, replenishes the oxygen vacancies, and restores the catalyst to its initial state, completing a catalytic cycle and achieving the efficient oxidation of VOCs [67,68]. This is consistent with the mechanism of the Mars–van Krevelen model. Moreover, by controlling the preparation conditions or doping specific elements, the concentration and distribution of oxygen vacancies in perovskite catalysts can be regulated, thereby optimizing their catalytic performance.

### 2.3. Preparation Methods

#### 2.3.1. Common Preparation Methods

##### Sol-Gel Method

The sol-gel method uses metal inorganic salts or metal alkoxides as precursors. After dissolving them, complexing agents are added, the pH value is adjusted, and other means are adopted to promote the complexation reaction between the metal ions in the solution and the complexing agents, forming a sol. In the sol system, the metal ions are wrapped by the complex agents to form stable sol particles. As the reaction proceeds, the sol particles are gradually connected through a polycondensation reaction to form a three-dimensional network structure of the gel. Finally, the gel is dried and calcined and other treatments are carried out to remove the organic components and water in it; then, the perovskite-type catalyst can be obtained [69].

This method enables the uniform mixing of metal ions at the molecular level, ensuring the uniformity of the catalyst composition, which allows the subsequent reaction to proceed more uniformly and reduces the possibility of composition segregation. During the formation process of the gel, a rich pore structure will be formed. These pores can provide more active sites and increase the contact area between the reactants and the catalyst. The catalyst prepared by the sol-gel method has a high specific surface area and good pore structure, which is conducive to the adsorption and diffusion of reactant molecules on the surface of the catalyst, thus improving the catalytic activity. Although the preparation process is relatively simple and the reaction conditions are mild and easy to control, there are also some disadvantages. For example, a large amount of organic reagents is required in the preparation process. These reagents are expensive, and they also generate a large amount of waste gas during the calcination process, causing pollution to the environment [70].

##### Co-Precipitation Method

The co-precipitation method involves mixing a mixed solution containing A-site and B-site metal ions with precipitants such as sodium carbonate, sodium hydroxide, oxalic acid, etc., so that the metal ions are simultaneously precipitated in the form of hydroxides, carbonates, or oxalates. During the precipitation process, the A-site and B-site metal ions are uniformly precipitated together according to the stoichiometric ratio to form a precursor precipitate. After the precursor precipitate is treated by filtration, washing, drying, calcination, and other processes, the perovskite-type catalyst can be obtained [71].

The co-precipitation method has a significant impact on the crystal structure and performance of the catalyst. By controlling the precipitation conditions, such as the type, concentration, dropping rate of the precipitant, reaction temperature, pH value, etc., the particle size, morphology, and composition uniformity of the precursor precipitate can be adjusted, thereby affecting the crystal structure and performance of the final catalyst [72]. Precipitating at a lower pH value may result in a precursor precipitate with smaller particles and a larger specific surface area, thus preparing a catalyst with higher catalytic activity. While precipitating at a higher pH value may lead to a precursor precipitate with larger particles and better crystallinity, endowing the catalyst with better thermal stability [73], it is worth noting that the precipitation sequence and rate of metal ions during the precipitation process will also affect the composition uniformity of the catalyst. If there is a large difference in the precipitation rates of A-site and B-site metal ions, it may lead to an uneven distribution of metal ions in the precipitate, thus affecting the performance of the catalyst [74]. The preparation process of the co-precipitation method is relatively simple and has a low cost, making it suitable for large-scale industrial production [75]. Moreover, compared with the sol-gel method, the co-precipitation method does not require the use of a large amount of expensive organic reagents, and the preparation cycle is relatively short. However, this method also has certain limitations. During the precipitation process, impurity ions are likely to be introduced, and these impurity ions may affect the performance of the catalyst [76].

##### Hydrothermal Synthesis Method

The hydrothermal synthesis method is used to make metal salts and other reactants undergo a chemical reaction under high-temperature and high-pressure conditions to directly generate perovskite-type crystals. In the hydrothermal reaction system, the high-temperature and high-pressure environment can promote the dissolution of reactants and the migration of ions, and accelerate the chemical reaction rate, which is conducive to the formation of perovskite-type crystals [77]. The hydrothermal synthesis method can be used to prepare perovskite catalysts with high crystallinity and good purity. Moreover, by regulating reaction conditions such as temperature, pressure, reaction time, and the pH value of the solution, the crystal structure and morphology of the catalyst can be controlled. However, this method has high requirements for equipment, and it is difficult to monitor and control the reaction process in real time [78].

#### 2.3.2. Advanced Preparation Methods

##### High-Energy Ball Milling Method

The high-energy ball milling method (HEBM) can usually process materials in a relatively simple way. It can more mechanistically process large-particle materials into small-particle materials, thereby significantly increasing the specific surface area of the materials [79]. Heidinger et al. prepared the LaCoO_3_ perovskite catalyst by a three-step reactive milling method, in which the high-energy ball milling method was the second step [80]. First, a solid-state reaction (SSR) was carried out. La_2_O_3_ and Co_3_O_4_ were uniformly mixed in a molar ratio of 1:0 and calcined at 1100 °C in a static air atmosphere for 4 h to obtain the perovskite phase. Then, HEBM was carried out. Nine grams of the SSR sample was taken, and a grinder, stainless steel equipment, and grinding balls were used. The sample was ground at a rotation speed of 1060 cycles·min^−1^ for 90 min in a static air atmosphere. Although the high-energy ball milling process can transform large particles of LaCoO_3_ into small particles, particle aggregation is likely to occur. Therefore, low-energy ball milling was finally carried out to optimize the dispersion of the sample particles, and the final sample was obtained. The performance of the sample (LaCo_HEBM) obtained by the high-energy ball milling method was significantly higher than that of the untreated sample (LaCo_SSR). This is mainly because the high-energy ball milling method significantly reduced the particle size of the material and increased its specific surface area. The sample (LaCo_LEBM60) that was treated by high-energy ball milling and then by low-energy ball milling for 60 min showed even better performance, which is attributed to the fact that the low-energy ball milling dispersed the aggregated small particles.

Compared with some traditional preparation methods of perovskite catalysts, the high-energy ball milling method does not require overly complex reactions. It is convenient to form smaller particles, and it can effectively increase the specific surface area of the catalyst and further improve its performance in the catalytic oxidation of VOCs. This method has a high degree of automation, can be produced on a large scale, has a low operation cost, and is suitable for industrial production.

##### Template Method

The template method utilizes the spatial confinement and structure-directing effects of the template agent to control the growth and assembly of the perovskite precursor, thereby precisely regulating the morphology, size, and pore structure of the catalyst [81]. Templates are divided into hard templates (such as mesoporous silica, alumina, and carbon nanotubes) and soft templates (such as surfactants, polymers, and biomolecules). Hard templates can provide stable spatial confinement, which is beneficial for the preparation of catalysts with ordered pore structures. Soft templates, on the other hand, have the advantages of a simple preparation process, low cost, and flexible regulation of morphology and size [82]. During the preparation, the template and the precursor solution are first prepared separately; then, the two are combined; finally, the precursor is formed into a perovskite structure, and the template is removed through heat treatment.

Compared with common perovskite preparation methods, such as the co-precipitation method, hydrothermal synthesis method, and sol-gel method, the template method has the advantage of being able to more precisely control the morphology, size, and pore structure of the perovskite, which can be achieved by selecting different types of templates, thus obtaining highly uniform and reproducible products. The perovskite prepared by this method often has a large specific surface area and a rich pore structure, which is conducive to the diffusion of substances and the exposure of more active sites, thereby improving the catalytic performance. At the same time, the template method has strong designability and can flexibly adjust the preparation conditions according to different requirements to obtain perovskite catalysts with specific structures and properties [75,83].

In a word, the common preparation methods include the sol-gel method, the co-precipitation method, and the hydrothermal synthesis method. The sol-gel method uses metal inorganic salts or metal alkoxides as precursors. After dissolution, complexing agents are added, the pH value is adjusted, etc., to form a sol. Then, through a polycondensation reaction, a gel is formed. Finally, after treatments such as drying and calcination, the perovskite-type catalyst is obtained. This method enables the uniform mixing of metal ions at the molecular level; the catalyst has a high specific surface area and a good pore structure. However, a large number of organic reagents are required in the preparation process, which is likely to cause environmental pollution. The co-precipitation method involves mixing a mixed solution containing A-site and B-site metal ions with precipitants, so that the metal ions are precipitated simultaneously to form a precursor precipitate, which is then processed to obtain the catalyst. This method can adjust the characteristics of the precursor precipitate by controlling the precipitation conditions, thereby affecting the performance of the catalyst. The preparation process is simple and low in cost, making it suitable for large-scale industrial production. However, impurity ions are likely to be introduced during the precipitation process, which may affect the performance of the catalyst. The hydrothermal synthesis method is used to directly generate perovskite-type crystals by making metal salts and other reactants undergo a chemical reaction under high-temperature and high-pressure conditions. It can be used to prepare perovskite catalysts with high crystallinity and high purity. The crystal structure and morphology of the catalyst can be controlled by adjusting the reaction conditions. However, this method has high requirements for equipment, and it is difficult to monitor and control the reaction process in real time. With the continuous development of perovskite catalysts, some advanced preparation methods have gradually been widely used by scientists, such as the high-energy ball milling method or the template method. People usually have a high degree of purpose when it comes to these advanced preparation methods. For example, they aim to obtain materials with a larger specific surface area or materials with certain specific morphologies, to better enhance the performance of the catalysts. Different preparation methods have their own advantages and disadvantages. In practical applications, it is necessary to select an appropriate preparation method according to specific needs and conditions to obtain perovskite catalysts with excellent performance.

### 2.4. Nanostructures and Morphologies

#### 2.4.1. Nanofibers

**Figure 4:**
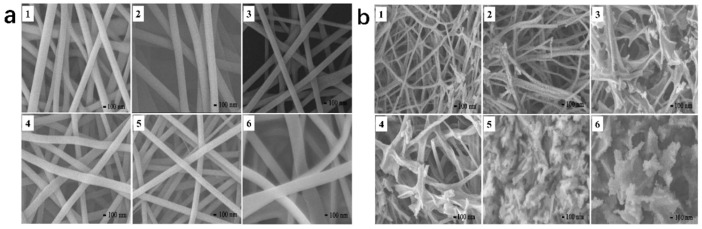
SEM images of (1) LaCoO_3−δ_, (2) La_0.8_Ce_0.2_CoO_3−δ_, (3) La_0.6_Ce_0.4_CoO_3−δ_, (4) La_0.4_Ce_0.6_CoO_3−δ_, (5) La_0.2_Ce_0.8_CoO_3−δ_, and (6) CeCoO_3−δ_: (**a**) electrospun nanofibers and (**b**) catalysts calcined at 600 °C. Reproduced with permission (Reproduced with permission from reference [84]).

Nanostructuring is capable of efficiently enhancing the specific surface area of the catalyst, thereby increasing the number of active sites [75]. Electrospinning has been extensively applied for the preparation of perovskite catalysts with nanofiber morphologies. Luo et al. synthesized La_1−x_Ce_x_CoO_3−δ_ (x = 0, 0.2, 0.4, 0.6, 0.8, 1.0) perovskites through an electrospinning route followed by a calcination process [84], The morphologies of these samples prior to and subsequent to the heat treatment are illustrated in Figure 4a,b. The perovskite nanofibers prepared by electrospinning are long and continuous and have smooth surfaces, with diameters ranging from 150 to 350 nanometers. After calcination, as shown in Figure 4b, the morphologies of these samples changed to varying degrees. Some nanoparticles can be clearly observed in the LaCoO_3−δ_ and La_0.8_Ce_0.2_CoO_3−δ_ nanofibers, exhibiting comparatively high specific surface area. Both La_0.6_Ce_0.4_CoO_3−δ_ and La_0.4_Ce_0.6_CoO_3−δ_ exhibit a duck-claw-like morphology, while CeCoO_3−δ_ shows a layered morphology. In addition, the morphology of La_0.2_Ce_0.8_CoO_3−δ_ is between those of La_0.4_Ce_0.6_CoO_3−δ_ and CeCoO_3−δ_. For the oxidation reaction of benzene, due to the increase in the specific surface area and the good preservation of the nanofiber morphology, the doping of Ce improves the catalytic activity of LaCoO_3_.

Luo et al. also prepared LaCoO_3_ for the benzene oxidation reaction by the SBA-15 (Santa Barbara Amorphous—15)-assisted electrospinning method [85]. It was found that the electrospinning technique is beneficial for the exposure of surface Co elements, which are the active sites for benzene oxidation. The electrospun LaCoO_3_ that has undergone alkali treatment exhibits greater catalytic activity towards benzene oxidation compared to the initial electrospun LaCoO_3_. This is due to the presence of a larger quantity of adsorbed oxygen species and exposed Co^3+^ on its surface, along with the generation of La(OH)_3_ nanosheets. Conversely, the electrospun LaCoO_3_ nanorods that have undergone alkali treatment for 9 h possess lower catalytic activity for the oxidation of benzene than the untreated LaCoO_3_. The reason behind this is that the excessive formation of La(OH)_3_/La_2_O_3_ species results in a reduction in the number of surface-active Co species [86]. The electrospun LaCoO_3_ after 3 h alkali treatment shows the highest catalytic activity for benzene oxidation, because a good balance is achieved between the amounts of La(OH)_3_/La_2_O_3_ and the surface-active Co species.

#### 2.4.2. 3D Ordered Macroporous (3DOM)

**Figure 5:**
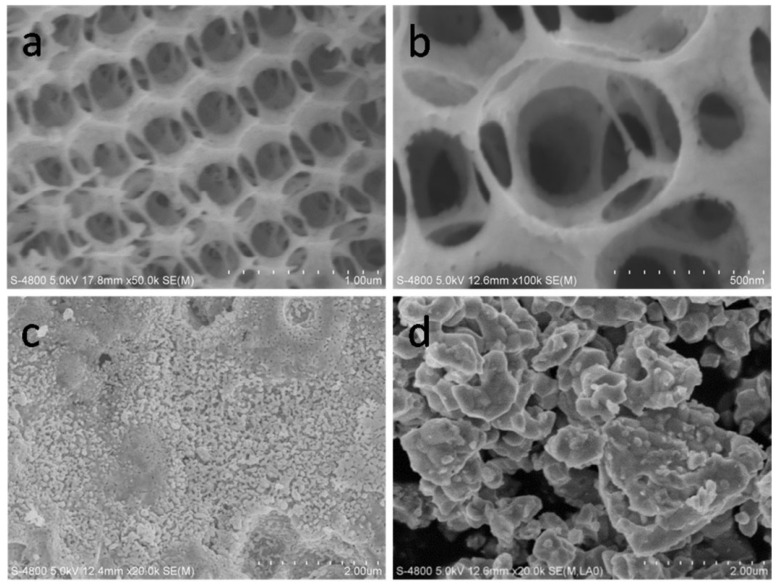
SEM images of (**a**,**b**) 3D-SC-800 sample, (**c**) 3D-SC-200 sample, and (**d**) SC sample (Reproduced with permission from reference [87]).

The template method is an effective way to prepare three-dimensionally ordered macroporous (3DOM) perovskite oxides. He et al. prepared 3DOM morphology materials through the polymethyl methacrylate (PMMA) colloidal crystal template method [87]. Firstly, hexagonal close-packed and ordered PMMA microspheres with averages diameters of approximately 800 nm and 200 nm were synthesized according to the method in the literature. The amount of 0.025 mM of Sm(NO_3_)_3_·6H_2_O and Co(NO_3_)_2_·6H_2_O (with a final total metal ion concentration of 2 M) was dissolved in 5 mL of ethylene glycol; then, methanol and EG were added to prepare a solution with a specific concentration, and 2.5–3.0 g of the PMMA colloidal crystal was immersed in this solution for 3–5 h. After that, the excess solution was removed by vacuum filtration, and it was dried at room temperature. Then, it was heated in a muffle furnace at a rate of 1 °C/min to 600 °C and maintained at this temperature for 5 h. 3DOM-SmCoO_3_ (3D-SC-800 and 3D-SC-200), synthesized using 800 nm and 200 nm PMMA templates, was obtained, respectively. At the same time, SmCoO_3_ (SC) was also prepared by the EDTA-citrate complex sol-gel method as a comparison. Through SEM observation, 3D-SC-800 (Figure 5a,b) exhibited an ordered 3DOM structure, with interconnected hexagonal pores and hemispherical voids. The pore diameter was 400–600 nm, the pore channels were 200–300 nm, and the pore walls were composed of linearly fused SmCoO_3_ nanosegments, along with 20–50 nm nanovoids. In Figure 5c, no macroscopic porous structure is observed in 3D-SC-200. And SC (Figure 5d) presents the morphology of conventional bulk crystalline oxide particles, with a limited specific surface area.

The above content focuses on the nanostructures and morphologies of perovskite catalysts, covering two major types: nanofibers and three-dimensionally ordered macroporous (3DOM) structures. In terms of nanofibers, electrospinning is a commonly used method for preparing perovskite nanofiber catalysts. Luo et al. successfully synthesized a series of La_1−x_Ce_x_CoO_3−δ_ perovskites through this method and subsequent calcination treatment. The prepared nanofibers have a diameter in the range of 150–350 nanometers; they are long and continuous and have smooth surfaces. After calcination at 600 °C, their morphologies change. For example, nanoparticles appear in the LaCoO_3−δ_ and La_0.8_Ce_0.2_CoO_3−δ_ nanofibers, increasing the specific surface area. Samples with different compositions exhibit unique morphologies such as duck-claw-like and layered shapes. Among them, the doping of Ce enhances the catalytic activity of LaCoO_3_ for the oxidation of benzene due to the increase in specific surface area and the maintenance of the fiber structure. In addition, for the LaCoO_3_ prepared by the SBA-15-assisted electrospinning method, after alkali treatment, the adsorbed oxygen species and the exposed Co^3+^ on the surface increase, and La(OH)_3_ nanosheets are generated simultaneously. The sample treated with alkali for 3 h shows the best catalytic activity for the oxidation of benzene, while the sample treated with alkali for 9 h has reduced activity for the oxidation of benzene compared to the untreated LaCoO_3_ because the excessive formation of La(OH)_3_/La_2_O_3_ reduces the number of surface-active Co species. In terms of the 3DOM structure, He et al. used the PMMA colloidal crystal template method to prepare perovskite oxides with a 3DOM morphology. First, PMMA microspheres with average diameters of approximately 800 nm and 200 nm were synthesized and combined with a solution prepared by dissolving Sm(NO_3_)_3_·6H_2_O and Co(NO_3_)_2_·6H_2_O to a total metal ion concentration of 2 M. 3DOM-SmCoO_3_ (3D-SC-800 and 3D-SC-200) was obtained using 800 nm and 200 nm PMMA templates, respectively. By comparing with SmCoO_3_ (SC) prepared by the EDTA-citrate complex sol-gel method, it was found that 3D-SC-800 exhibits an ordered 3DOM structure with interconnected hexagonal pores and hemispherical voids. The pore diameter is 400–600 nm, the pore channels are 200–300 nm, and the pore walls are composed of linearly fused SmCoO_3_ nanosegments with 20–50 nm nanovoids. 3D-SC-200 has no macroscopic porous structure, and SC has the morphology of conventional bulk crystalline oxide particles with a limited specific surface area.

### 2.5. Modification of Perovskite Catalysts

#### 2.5.1. Doping of Metal Ions

In the modification research of perovskite catalysts, the doping of metal ions is a commonly used and effective method. By introducing different metal ions at the A-site or B-site, it can have a significant impact on the crystal structure, electronic properties, and surface properties of the catalyst, thereby optimizing its performance in the catalytic oxidation removal of VOCs [88,89].

##### A-Site Doping

The A-site cations do not directly participate in the catalytic oxidation reaction in principle. However, they may affect the catalytic activity of perovskite oxides by influencing the electronic structure, defect structure, and surface properties of the B-site cations [46]. When the oxidation state of the B cation increases, the redox process becomes relatively easy, and more oxygen species can be generated at low temperatures, thereby enhancing the overall oxidation activity [90]. In addition, oxygen vacancies are beneficial to the catalytic activity in the oxidation reaction because they increase the mobility of lattice oxygen [91]. At present, many A-site partially substituted perovskite catalysts (A_x_A’_1−x_BO_3_) exhibit excellent catalytic oxidation performance for VOCs.

**Figure 6:**
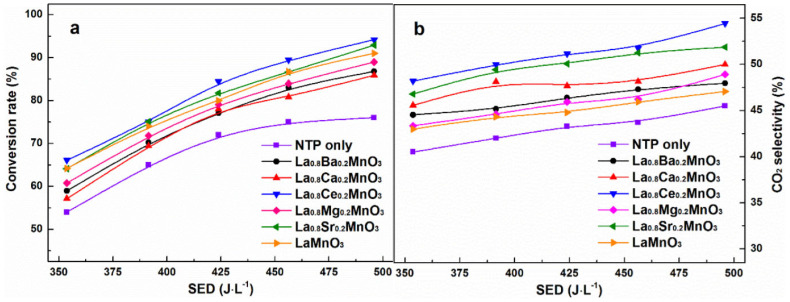
(**a**) Conversion rate of ethyl acetate and (**b**) selectivity of CO_2_ of La_0.8_M_0.2_MnO_3_ (M = Ba, Ca, Ce, Mg and Sr) catalysts (Reproduced with permission from reference [92]).

**Figure 7:**
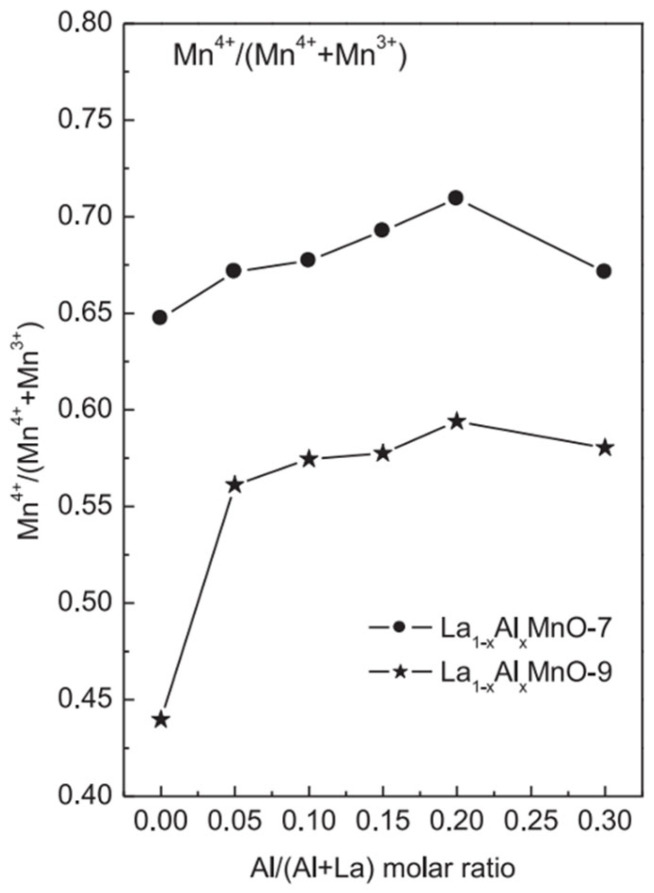
The molar ratio of Mn^4+^/(Mn^4+^ + Mn^3+^) in Al_x_La_1−x_MnO-7 and Al_x_La_1−x_MnO-9 catalysts (Reproduced with permission from reference [93]).

Among perovskite catalysts, LaMnO_3_ has received extensive attention due to its thermal stability, the mixed valence state of the transition metals, and its oxygen migration ability and has become the basic catalyst for doping modification. Zhu et al. synthesized a series of A-site-substituted La_0.8_M_0.2_MnO_3_ catalysts (M = Ba, Ca, Ce, Mg and Sr) by the sol-gel method and conducted catalytic oxidation tests of ethyl acetate in a dielectric barrier discharge reactor [92]. Compared with pure LaMnO_3_, the La_0.8_M_0.2_MnO_3_ catalysts exhibit higher adsorbed oxygen content, and only doping with Ce and Sr can improve the reducibility of the catalysts; as shown in Figure 6, among these catalysts, La_0.8_Ce_0.2_MnO_3_ shows the highest ethyl acetate conversion and CO_2_ selectivity, indicating that the reducibility of the catalyst plays an important role in determining the reaction performance of the plasma catalytic oxidation process. In LaMnO_3_, using Al to substitute the A-site cations is also beneficial in improving the catalytic performance. Chen et al. prepared a series of aluminum-substituted La_1−x_Al_x_MnO_3_ (x = 0–0.3) catalysts by the sol-gel method [93]. The improvement in reducibility is conducive to the activation of oxygen species, and the high surface acidity can promote the chemisorption of 1,2-dichloroethane. The synergy of both leads to the enhanced catalytic activity of La_1−x_Al_x_MnO_3_. It can be observed in Figure 7 that doping Al in the catalyst increases the content of high-valent Mn^4+^ substances, which is closely related to the enhancement of the reducibility of the catalyst.

##### B-Site Doping

**Figure 8:**
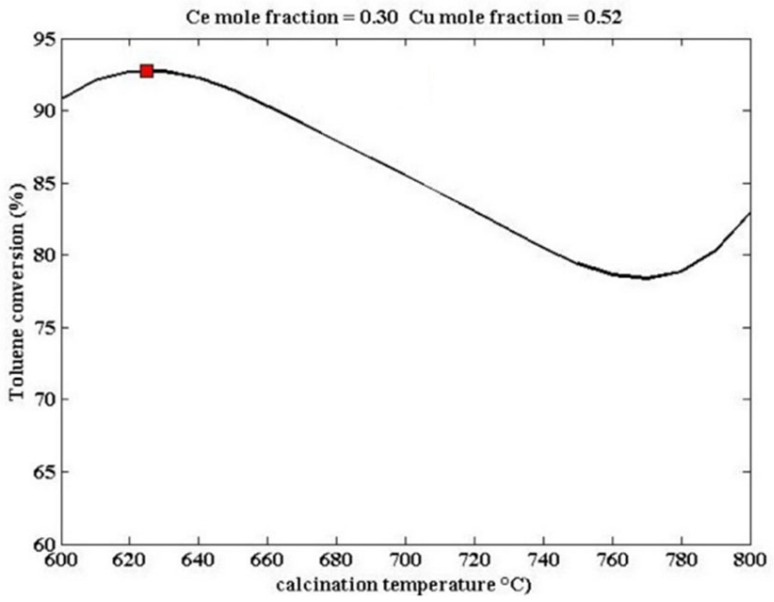
The influence of catalyst design parameters on La_1−x_Ce_x_Mn_1−y_Cu_y_O_3_ (x = 0.30, y = 0.52) (Reproduced with permission from reference [94]).

The doping modification of perovskite catalysts is not limited to the partial substitution at the A-site. The partial substitution of B-site cations can also improve catalytic activity. As shown in Figure 8, Zonouz et al. combined artificial neural networks with genetic algorithms to find the optimal values of catalyst design parameters for catalytic oxidation of toluene by studying the mole fractions of Ce and Cu and the calcination temperature [94]. At 625 °C, the conversion rate of toluene over La_1−x_Ce_x_Mn_1−y_Cu_y_O_3_ (x = 0.30, y = 0.52) is the highest, reaching approximately 92.1% (marked with red dot), demonstrating that the introduction of Ce and Cu into the perovskite structure can improve the reduction ability of the catalyst.

Meanwhile, the partial substitution of elements at the B-site is not limited to La-based perovskite catalysts. Rezlescu et al. prepared SrMn_1−x_Ce_x_O_3_ (x = 0, 0.2) perovskite catalysts by the self-propagating combustion method [95]. These catalysts were calcined at 1000 °C for 4 h, and the catalytic performance of the perovskite nanopowders was tested through the catalytic combustion of dilute acetone under normal pressure. In the perovskite structure of SrMnO_3_, the partial substitution of Mn by Ce ions (x = 0.2) leads to smaller microcrystalline sizes, a larger specific surface area, and the presence of Ce and Mn cations with variable valence states in the perovskite structure, which significantly improves the catalytic activity of the perovskite. When SrMn_0.8_Ce_0.2_O_3_ is used for the catalytic combustion of low-concentration acetone (with a concentration of 1–2‰ in the air), the conversion rate of acetone exceeds 90% at 200 °C, while the conversion rate of SrMnO_3_ is only 50%.

##### A- and B-Site Co-Doping

**Figure 9:**
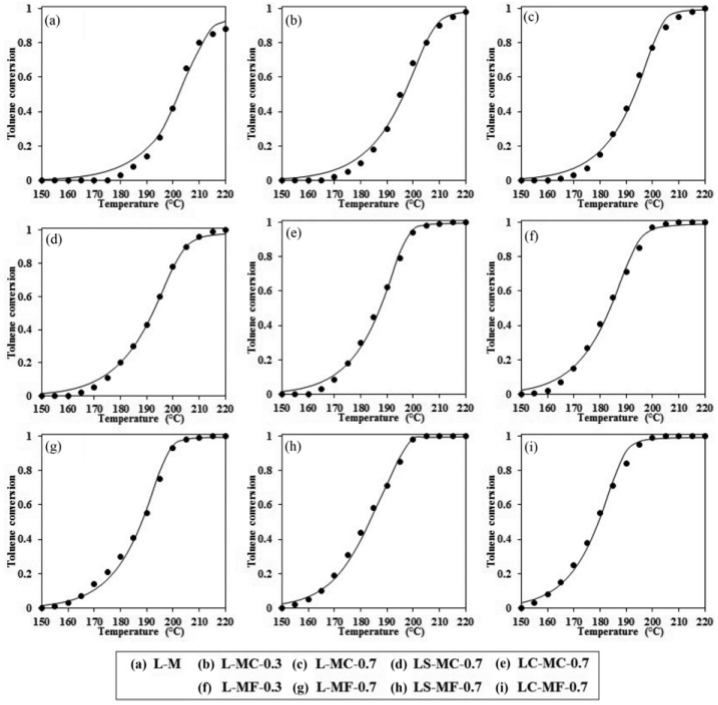
Toluene conversion rate of the LH-OS-D model within the reaction temperature range (Reproduced with permission from reference [96]).

Co-doping at the A- and B-sites can simultaneously achieve the structural adjustment of the catalyst and the electronic modification of the perovskite oxide. Ali et al. synthesized La_0.8_A_0.2_Mn_0.3_B_0.7_O_3_ (A = Sr, Ce; B = Cu, Fe) by the sol-gel auto-combustion method [96]. After Mn is partially replaced by other elements, the oxygen vacancies of the catalyst increase, thus changing its surface structure and improving its reducibility. The partial substitution of Sr and Ce at the A-site of the perovskite catalyst also enhances their activity for the oxidation of toluene. The introduction of Sr^2+^ and Ce^4+^ at the A-site of the perovskite increases the relative contents of Cu^2+^, Fe^3+^, Mn^4+^, and O_ads_ in the catalyst, and changes the reducibility of the cations at the B-site. These factors will cause defects in the perovskite structure, leading to higher catalytic performance. As can be observed in Figure 9, for the catalytic oxidation of toluene, the iron-containing perovskite catalyst exhibits higher activity than the copper-containing perovskite. Among them, La_0.8_Ce_0.2_Mn_0.3_Fe_0.7_O_3_ has the highest activity, and this catalyst achieves a 100% toluene removal rate at 200 °C.

#### 2.5.2. Supported Catalysts

**Figure 10:**
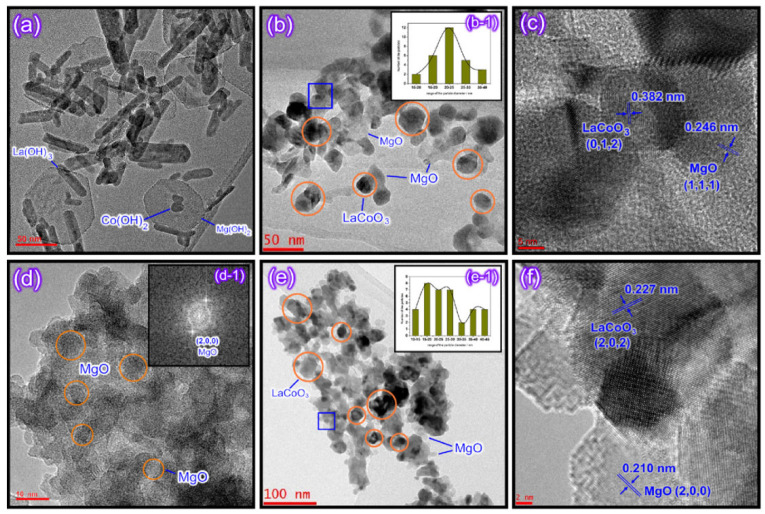
HR-TEM images of (**a**) A-sc-LCM sample, (**b**) sc-LCM sample, (**d**) A-cr-LCM sample and (**e**) cr-LCM sample; (**b-1**) and (**e-1**) represent the corresponding particle size distribution; (**d-1**) shows a fast Fourier transform pattern of MgO in the A-cr-LCM sample; (**c**,**f**) show the d-spacing of LaCoO_3_ and MgO in the sc-LCM and cr-LCM samples, respectively. (Reproduced with permission from reference [97]).

Zhang et al. synthesized LaCoO_3_/MgO composite perovskites by the methods of supercritical water and citrate, respectively [97]. The sample prepared by the supercritical water method is shown in Figure 10. The LaCoO_3_ particles are in full contact with the MgO support, resulting in the generation of more oxygen vacancies and abundant weakly chemisorbed oxygen in the structure. Therefore, it exhibits good catalytic performance in the oxidation of toluene and methane.

**Figure 11:**
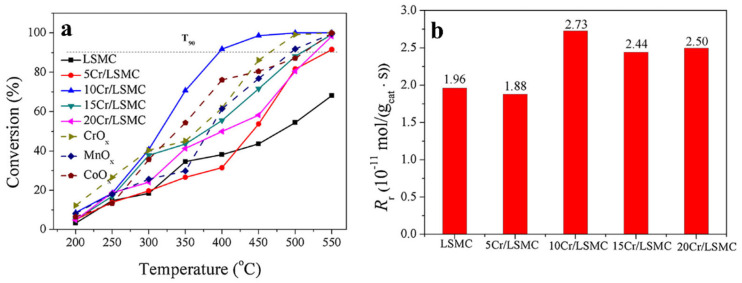
(**a**) Catalytic activity of the synthesized catalysts for the oxidation of 1,2-DCE, (**b**) oxidation rate of 1,2-DCE at 250 °C (Reproduced with permission from reference [98]).

Tian et al. synthesized CrO_x_/LaSrMnCoO_6_ catalysts with different loadings, enabling LaSrMnCoO_6_ to combine the advantages of the high activity and chlorine resistance that CrO_x_ possesses [98]. This catalyst was used for the first time in the deep oxidation of 1,2-dichloroethane. As shown in Figure 11a, due to the fact that the 10 wt.% CrO_x_/LaSrMnCoO_6_ (10Cr/LSMC) catalyst has a relatively large number of Cr^6+^ species, high reducibility, excellent adsorption capacity for 1,2-dichloroethane, and a large amount of surface-active lattice oxygen species, and the presence of Cr^6+^ promotes the interaction between the surface lattice oxygen and LaSrMnCoO_6_, 90% of 1,2-dichloroethane is completely decomposed at 400 °C. Similarly, Figure 11b also shows that the reaction rate of 10Cr/LSMC is the highest. During the oxidation of 1,2-dichloroethane, the main reaction by-products detected are vinyl chloride, 1,1,2-trichloroethane, trichloroethylene, and perchloroethylene, but they can be completely oxidized into CO, CO_2_, H_2_O, HCl, and Cl_2_. The introduction of CrO_x_ into LaSrMnCoO_6_ accelerates the oxidation process of 1,2-dichloroethane and reduces the generation of by-products. At the same time, it also shows excellent durability and water resistance.

It is worth noting that the modification with noble metals can increase the storage and mobility of oxygen in perovskite. Therefore, modifying perovskite by loading noble metals is also one of the effective ways to improve the catalytic activity of perovskite catalysts for the oxidation of VOCs. The catalytic performance of the supported gold is related to factors such as the size of the gold nanoparticles, the properties of the support, and the preparation method. Generally speaking, gold particles can exhibit high catalytic activity when their size is less than 5 nm [99]. Li et al. prepared rhombohedral crystalline three-dimensional ordered macroporous (3DOM) LaCoO_3_ and its supported catalyst Au/3DOM LaCoO_3_ and evaluated their catalytic activities for the oxidation of toluene and CO [100]. The Au/3DOM LaCoO_3_ sample exhibited a 3DOM structure and had a high specific surface area of 24–29 m^2^/g. The Au nanoparticles were well dispersed on the walls of 3DOM LaCoO_3_, with an average particle size of 2–4 nm. The strong interaction between Au and LaCoO_3_ leads to the transfer of lattice oxygen or adsorbed oxygen on or within the support to the support vacancies at low temperatures, increasing the content of surface-adsorbed oxygen. The increase in surface-active oxygen species has a great impact on the improvement of the deep oxidation performance. The increase in the Au loading also increases the concentration of Au^δ⁺^ species (such as Au^1+^ and Au^3+^) that are more active than Au°. The high oxygen ion concentration and good low-temperature reducibility and durability, as well as the strong interaction between Au and 3DOM LaCoO_3_, enable the 7.63 wt% Au/3DOM LaCoO_3_ to exhibit the best catalytic performance.

This part of the content mainly introduces the modification methods of perovskite catalysts, including two categories: metal ion doping and supported catalysts. In terms of metal ion doping, although A-site doping does not directly participate in the catalytic oxidation reaction, it can affect the electronic structure of B-site cations, etc., thereby influencing the catalytic activity. For example, among the A-site-substituted La_0.8_M_0.2_MnO_3_ (M = Ba, Ca, Ce, Mg and Sr) catalysts prepared by Zhu et al. through the sol-gel method, La_0.8_Ce_0.2_MnO_3_ has the highest conversion rate of ethyl acetate and selectivity for CO_2_. The aluminum-substituted La_1−x_Al_x_MnO_3_ (x = 0–0.3) catalysts prepared by Chen et al. have enhanced catalytic activity due to the improved reducibility and increased surface acidity. B-site doping can also improve catalytic activity. Zonouz et al. found through research that at 625 °C, the conversion rate of toluene over La_1−x_Ce_x_Mn_1−y_Cu_y_O_3_ (x = 0.30, y = 0.52) is the highest, reaching approximately 92.1%. In the SrMn_1−x_Ce_x_O_3_ (x = 0, 0.2) catalysts prepared by Rezlescu et al., when x = 0.2, the conversion rate of low-concentration acetone (with a concentration of 1–2‰ in the air) at 200 °C exceeds 90%, while that of the undoped SrMnO_3_ is only 50%. Co-doping at both the A- and B-sites can achieve the structural adjustment of the catalyst and the electronic modification of the perovskite oxide simultaneously. Among the La_0.8_A_0.2_Mn_0.3_B_0.7_O_3_ (A = Sr, Ce. B = Cu, Fe) synthesized by Ali et al., La_0.8_Ce_0.2_Mn_0.3_Fe_0.7_O_3_ can achieve a 100% toluene removal rate at 200 °C. In terms of supported catalysts, the LaCoO_3_/MgO composite perovskite prepared by Zhang et al. shows good catalytic performance in the oxidation of toluene and methane because the LaCoO_3_ particles are in full contact with the MgO support, generating more oxygen vacancies and abundant weakly chemisorbed oxygen. The 10 wt.% CrO_x_/LaSrMnCoO_6_ catalyst synthesized by Tian et al. can decompose 90% of 1,2-dichloroethane at 400 °C and has excellent durability and water resistance. The 7.63 wt% Au/3DOM LaCoO_3_ prepared by Li et al. exhibits the best catalytic performance in the oxidation of toluene and CO due to the strong interaction between Au and LaCoO_3_. This sample has a specific surface area of 24–29 m^2^/g, and the average particle size of the Au nanoparticles is 2–4 nm. A more specific distinction is shown in Table 1, which is classified according to different catalyst systems.

## 3. Application

### 3.1. Photocatalysis

Perovskite materials exhibit remarkable advantages in the field of photocatalytic oxidation of VOCs due to their adjustable structures, strong light absorption capabilities, and efficient charge separation characteristics [101]. These materials generate electron-hole pairs upon photoexcitation, which react with oxygen and water molecules adsorbed on the surface to produce strongly oxidizing reactive oxygen species, such as ·OH and ·O^2−^ [102]. These reactive oxygen species can gradually degrade VOCs such as benzene and formaldehyde into CO_2_ and H_2_O. Compared with traditional catalysts, perovskites have a wider visible light response range (up to 800 nm), and their stability and quantum efficiency can be further improved through methods such as element doping (for example, alkali metals at the A-site and transition metals at the B-site) [103]. They are especially suitable for the efficient treatment of low-concentration VOCs.

**Figure 12:**
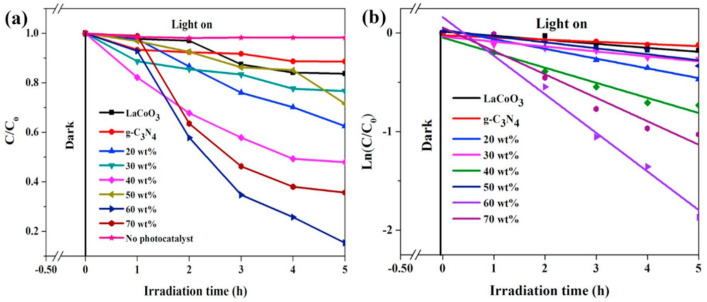
(**a**) Photocatalytic activity of LaCoO_3_, g-C_3_N_4_, and LaCoO_3_/g-C_3_N_4_ composite materials in the degradation of phenol under visible light irradiation; (**b**) Ln(C/C_0_) ~t curves of LaCoO_3_, g-C_3_N_4_, and LaCoO_3_/g-C_3_N_4_ composite materials (Reproduced with permission from reference [104]).

Jin et al. prepared a Z-scheme LaCoO_3_/g-C_3_N_4_ heterojunction photocatalyst through a one-step impregnation method and tested its performance in degrading phenol under visible light [104]. As shown in Figure 12a, the LaCoO_3_/g-C_3_N_4_-60 wt% composite catalyst exhibits the best activity, with a phenol degradation rate of 85% within 5 h, which is 5.2 times and 7.5 times that of pure LaCoO_3_ and g-C_3_N_4_, respectively. Meanwhile, the photocatalytic degradation process of phenol follows pseudo-first-order kinetics (Figure 12b). The figure shows that the content of g-C_3_N_4_ has a great influence on photocatalytic activity. A g-C_3_N_4_ content of 60 wt% is most favorable for the formation of more heterostructures between LaCoO_3_ and g-C_3_N_4_, which facilitates the separation of photogenerated charge carriers and enhances the photocatalytic activity.

However, perovskite photocatalysts also face some challenges in the application of the photocatalytic oxidation of VOCs. On the one hand, the recombination problem of photogenerated electron-hole pairs is relatively serious. During the migration process of photogenerated carriers inside and on the surface of the catalyst, electrons and holes are prone to recombine, which greatly reduces the number of effective carriers participating in the photocatalytic reaction, thus limiting the further improvement of the photocatalytic efficiency [105,106]. To solve this problem, researchers have adopted various methods, such as surface modification and construction of heterojunctions, to inhibit the recombination of photogenerated carriers and extend their lifetime. On the other hand, the utilization rate of visible light by most current perovskite photocatalysts still needs to be improved. Although methods such as ion doping can expand their response range to visible light to a certain extent, the overall utilization efficiency of solar energy is still low, and the advantages of photocatalytic technology cannot be fully utilized [107,108]. Therefore, developing new perovskite photocatalysts or optimizing the preparation process of existing catalysts to improve their absorption and efficient utilization of visible light is one of the important research directions at present.

### 3.2. Thermal Catalysis

Thermal catalytic oxidation is a widely used technology for the treatment of VOCs at present, and perovskite catalysts also exhibit unique properties in this field. In the process of thermal catalytic oxidation, VOC molecules are first adsorbed on the surface-active sites of the perovskite catalyst. Meanwhile, oxygen molecules are also adsorbed and activated. Under appropriate temperature conditions, the adsorbed VOC molecules react chemically with the activated oxygen species and are gradually oxidized into CO_2_ and H_2_O. Factors such as the nature and number of active sites of the catalyst, as well as the crystal structure of the catalyst, play a crucial role in the rate and selectivity of the thermal catalytic reaction [109,110,111].

Many perovskite catalysts exhibit high catalytic activity and can achieve efficient conversion of VOCs at relatively low temperatures. Taking the catalytic oxidation of toluene as an example, some doped perovskite catalysts can achieve a high conversion rate of toluene within the temperature range of 300–400 °C, which has obvious energy-saving advantages compared with traditional catalysts. Secondly, perovskite catalysts have good anti-poisoning properties against some common poisons such as sulfur and chlorine [112]. In actual industrial waste gases, there are often various impurities. Traditional noble metal catalysts are easily deactivated by these poisons, while perovskite catalysts can resist the erosion of poisons to a certain extent and maintain relatively stable catalytic activity, which makes them have greater application potential in the treatment of complex industrial waste gases [113].

**Figure 13:**
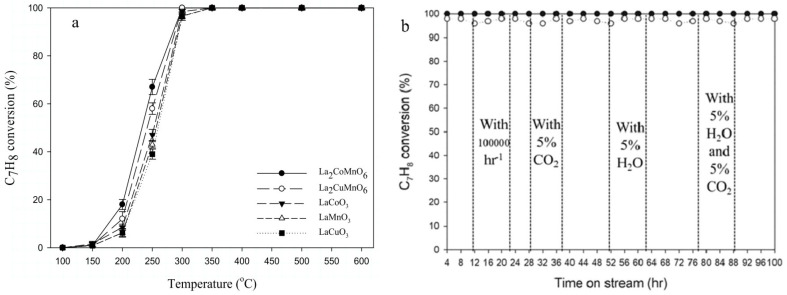
(**a**) Removal rate of C_7_H_8_, (**b**) durability test of the removal efficiency of C_7_H_8_ (Reproduced with permission from reference [114]).

Pan et al. prepared double perovskite catalysts, including La_2_CoMnO_6_ and La_2_CuMnO_6_, by the improved Pechini method [114]. In a fixed-bed reactor, using toluene and other VOCs as target pollutants, under different conditions such as temperature, gas hourly space velocity (GHSV), and the contents of CO_2_ and H_2_O, a Fourier transform infrared spectrophotometer and a CO_2_ analyzer were used to measure the concentrations of reactants and products, and the removal efficiency and CO_2_ yield were calculated to test their activity. The durability test was carried out at 300 °C with a GHSV of 30,000 h^−1^ and containing 5% CO_2_ and 5% H_2_O. The double perovskite catalysts have better activity and lower activation energy than single perovskite catalysts. The test results are shown in Figure 13; La_2_CoMnO_6_ can completely oxidize toluene into CO_2_ at 300 °C. In the durability test, La_2_CoMnO_6_ and La_2_CuMnO_6_ maintain a toluene removal efficiency of 100% and 98%, respectively.

**Figure 14:**
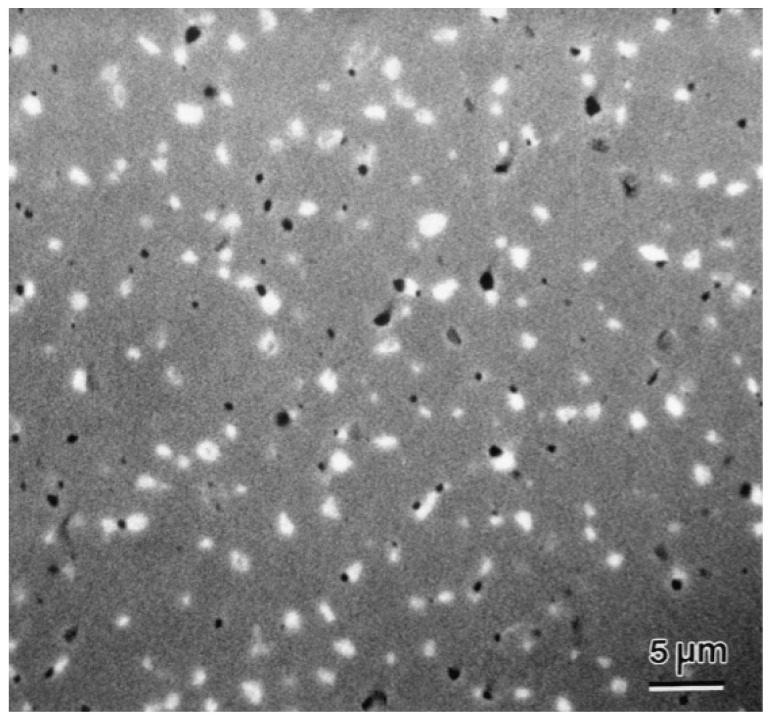
SEM of LaCoO_3_ sintered at 1350 °C (Reproduced with permission from reference [116]).

However, when the perovskite catalyst is used for a long time under high-temperature conditions, a sintering phenomenon may occur. Sintering will lead to a decrease in the specific surface area of the catalyst and a reduction in the number of active sites, thus gradually reducing the catalytic performance [115]. As shown in Figure 14, Kleveland et al. demonstrated the sintering phenomenon of LaCoO_3_ at 1350 °C. After sintering, the LaCoO_3_ particles fused with each other, forming larger particles and reducing the number of voids [116]. In order to overcome this problem, researchers usually adopt methods such as adding promoters and optimizing the preparation process to improve the anti-sintering performance of the catalyst. In addition, the reaction temperature window for the thermal catalytic oxidation of VOCs is relatively narrow. In order to obtain good catalytic activity and stability, the reaction temperature needs to be strictly controlled. This places high requirements in terms of the reaction equipment and operating conditions, increasing the difficulty and cost of practical applications [117].

### 3.3. Electrocatalysis

Electrocatalytic oxidation initiates an electrochemical reaction on the surface of the perovskite electrode by applying an external electric field, achieving the oxidative decomposition of VOCs [118]. In the electrocatalytic system, VOC molecules lose electrons and are oxidized on the anode surface, while a reduction reaction occurs at the cathode. Usually, oxygen gains electrons and is reduced to water or hydroxide ions. The presence of the electric field promotes the transfer of electrons and accelerates the oxidation process of VOCs, enabling the reaction to proceed under relatively mild conditions [119,120].

The electrocatalytic oxidation of VOCs has some unique advantages. Firstly, the reaction conditions are mild. Compared with thermal catalysis, electrocatalysis can achieve the effective conversion of VOCs at lower temperatures, which greatly reduces energy consumption and equipment costs [121]. Secondly, the electrocatalytic process is easy to regulate. By changing parameters such as the applied voltage and current density, the rate and selectivity of the electrocatalytic reaction can be conveniently adjusted to meet the treatment requirements of VOC waste gases with different types and concentrations [122]. This ability of precise regulation gives electrocatalytic oxidation technology great advantages in treating VOC waste gases with complex compositions.

**Figure 15:**
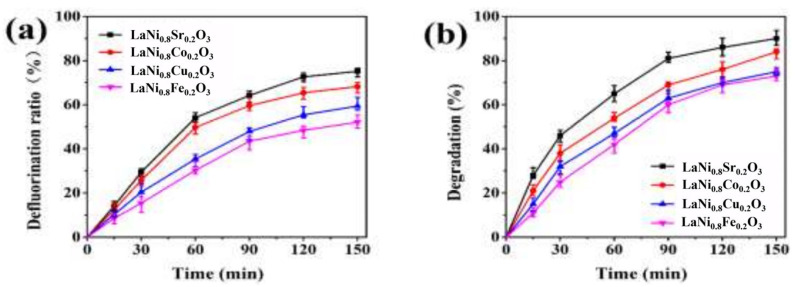
Defluorination rate curves (**a**) and PFOA degradation curves (**b**) of different catalytic electrodes (Reproduced with permission from reference [123]).

Chen et al. prepared LaNi_x_Y_1−x_O_3_ (Y = Fe, Cu, Co, Sr) catalysts by the sol-gel method and the citric acid complexation method and tested the electrocatalytic degradation performance of the perfluorooctanoic acid (PFOA) of the electrodes using cyclic voltammetry [123]. The amounts of 0.6 g of LaNi_x_Y_1−x_O_3_, 5.4 g of graphite, 1.0 g of Na_2_SO_4_, 0.6 g of acetylene black, and 3.0 g of PTFE were added to ethanol and heated to obtain aggregates. Subsequently, these aggregates were brought into contact with pretreated nickel foam. Finally, a gas diffusion electrode (GDE) was obtained through processes such as pressing, calcination, and soaking. In the electrochemical experiments, the GDE was used as the working electrode, and a common graphite electrode was used as the auxiliary electrode. Degradation experiments were carried out on the solution under specific conditions such as the electrolyte, current density, and aeration rate. At the same time, the current density, pH, and initial concentration of PFOA were changed to explore their impacts on the degradation effect. An ion chromatograph was used to determine the concentration of F^−^ to calculate the defluorination efficiency, and high-performance liquid chromatography–mass spectrometry was used to analyze the concentration of PFOA and the degradation products. The test results are shown in Figure 15. The LaNi_0.8_Sr_0.2_O_3_ catalyst particles are more uniformly dispersed, with higher current efficiency and stronger catalytic oxygen reduction ability. Under the conditions of a current density of 20 mA/cm^2^, pH of 5, and an initial concentration of 0.25 mmol/L, the degradation efficiency of PFOA by LaNi_0.8_Sr_0.2_O_3_ reaches 90.0%, the defluorination efficiency reaches 75.1%, and PFOA is finally degraded into F^−^, H_2_O, and CO_2_.

**Figure 16:**
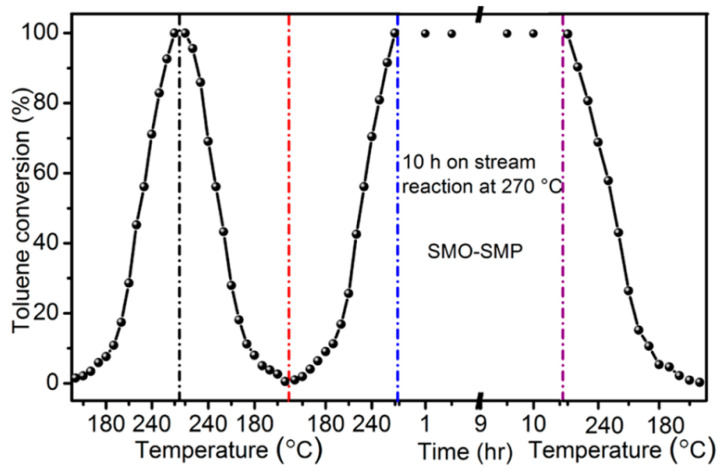
Stability of SMO-SMP at 270 °C and WHSV = 32,000 mL g^−1^ h^−1^ (Reproduced with permission from reference [126]).

However, the electrocatalytic oxidation technology of VOCs also faces some challenges. The stability of the electrode material is a key issue. During the electrocatalytic process, the perovskite electrode material may be corroded by the electrolyte solution, or its structure may change during long-term electrochemical reactions, thus affecting its service life and the stability of its catalytic performance [124]. In addition, the current energy efficiency of electrocatalytic oxidation of VOCs is relatively low, and a large amount of electrical energy is required to achieve the removal of VOCs [125]. This not only increases the treatment cost but also limits the large-scale application of this technology. To improve the stability of the electrode material and the energy efficiency, researchers have also carried out a large amount of research work. For example, Liu et al. prepared the SmMnO_3_ perovskite catalyst (SMO-SMP) by the self-fluxing polymerization method. Compared with the catalysts prepared by three common preparation methods, namely the co-precipitation method (SMO-CP), the sol-gel method (SMO-SG), and the impregnation method (SMO-IM), it has better reaction activity and excellent stability [126]. As shown in Figure 16, the stability of SMO-SMP was evaluated under the condition of long-term continuous operation with a weight hourly space velocity (WHSV) of 32,000 mL·g^−1^·h^−1^. Under this condition, it can be divided into three stages. The first stage is the cyclic test, and the activity test is gradually carried out from a low temperature to a high temperature. Second, starting from the highest point, the test is carried out by directly reducing the high temperature to the low temperature. Finally, the heating process is started again for the next test. It can be found that in the first stage, the differences of T_50_% and T_90_% are only 2 °C and 4 °C when changing from a low temperature to a high temperature, and only 2 °C and 3 °C when changing from a high temperature to a low temperature. This indicates that SMO-SMP has relatively good cyclic stability. When the temperature is raised to the highest temperature of 270 °C for the second time, it enters the second stage. A 10 h long-term activity test is carried out, and the toluene conversion rate hardly changes, which indicates that the catalyst has long-term stability. During the cooling test in the third stage, the activity of the catalyst is still good, further demonstrating that SMO-SMP has excellent stability.

### 3.4. Plasma-Assisted Catalysis

Plasma-assisted catalytic oxidation is a new method for treating VOCs that combines plasma technology with catalytic technology. In this system, the plasma discharge process will generate a large number of active species, such as high-energy electrons, ions, and free radicals; there is a strong synergistic effect between these active species and the perovskite catalyst [127,128,129]. On the one hand, the active species generated by the plasma can directly react with VOC molecules, oxidizing and decomposing them. On the other hand, these active species can also activate the surface of the perovskite catalyst, enhancing the catalyst’s ability to adsorb and catalytically oxidize VOCs, thus significantly improving the overall removal efficiency of VOCs [127].

**Figure 17:**
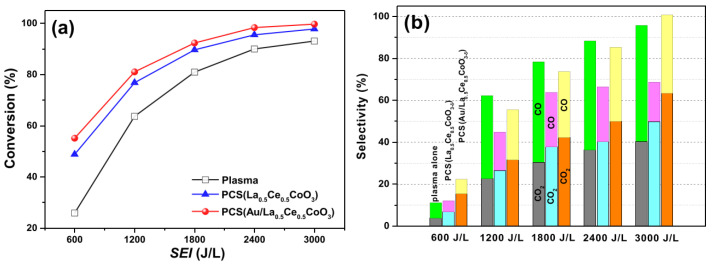
(**a**) XC_7_H_8_ as a function of SEI for different systems, (**b**) CO_x_ selectivity of different systems (Reproduced with permission from reference [130]).

Zhu et al. prepared the catalyst Au/La_1−x_Ce_x_CoO_3−δ_ through self-propagating high-temperature synthesis combined with an improved impregnation method [130]. Through plasma discharge, in the toluene oxidation and removal experiment, a self-made DBD (dielectric barrier discharge) reactor was used, with simulated air and toluene as the reaction gases, and the products were detected by a GC and a CO_x_ analyzer. The results, as shown in Figure 17, indicate that PCS(Au/La_0.5_Ce_0.5_CoO_3−δ_) has superior performance in toluene removal. It can completely convert toluene into CO_2_ (63%) and CO (37%) at an SEI of 3000 J/L and can inhibit the generation of O_3_ and NO_x_, showing stability during about 300 min of continuous reaction.

Plasma-assisted catalytic oxidation of VOCs has obvious advantages. This system can enhance the reaction effect. For some refractory VOCs, it is often difficult to achieve efficient removal when using plasma or a catalyst alone. However, in the plasma-assisted catalytic system, a high conversion rate can be achieved with a relatively low energy input. In addition, plasma-assisted catalysis can also broaden the applicable range of reactions. For some VOCs that are difficult to catalytically oxidize under conventional conditions, the high-energy effect of the plasma can make them more prone to reaction, thus expanding the application range of perovskite catalysts [131,132].

In summary, in the field of photocatalysis, perovskite catalysts, with their adjustable structures, strong light absorption capabilities, and efficient charge separation characteristics, exhibit remarkable performance in treating low-concentration VOCs. For instance, the LaCoO_3_/g-C_3_N_4_-60 wt% composite catalyst prepared by Jin et al. achieved an 85% degradation rate of phenol within 5 h, significantly outperforming single catalysts. However, issues such as severe recombination of electron-hole pairs and low utilization of visible light still exist. In thermal catalysis, many perovskite catalysts can efficiently convert VOCs at temperatures ranging from 300 to 400 °C. For example, La_2_CoMnO_6_ prepared by Pan et al. can completely oxidize toluene at 300 °C and shows strong resistance to sulfur and chlorine poisoning. Nevertheless, sintering is likely to occur at high temperatures, and the reaction temperature window is narrow. Electrocatalysis, driven by an external electric field, features mild reaction conditions and easy regulation. The LaNi_0.8_Sr_0.2_O_3_ catalyst prepared by Chen et al. achieved a 90.0% degradation rate of PFOA and a defluorination efficiency of 75.1% under specific conditions. However, it faces challenges such as poor stability of electrode materials and low energy efficiency. Plasma-assisted catalysis combines plasma and catalytic technologies. The Au/La_0.5_Ce_0.5_CoO_3−δ_ catalyst prepared by Zhu et al. could completely convert toluene into CO2 (63%) and CO (37%) at a specific energy input of 3000 J/L during the toluene oxidation and removal experiment, demonstrating excellent performance in treating refractory VOCs and expanding the applicable range of reactions. However, it may involve complex side reactions. Therefore, the optimal catalytic method can only be determined by comprehensively considering practical conditions such as the type and concentration of VOCs, treatment scale, energy consumption costs, and equipment requirements, to fully unleash the maximum efficiency of perovskite catalysts in the catalytic oxidation of VOCs.

## 4. Challenges and Prospects

### 4.1. Problems and Challenges

During the process of catalytic oxidation of VOCs by perovskite catalysts, catalyst deactivation is the primary problem. The phenomenon of carbon deposition will cause incompletely oxidized carbonaceous deposits to cover the active sites, hindering the progress of the reaction [133]. Sulfur-containing and chlorine-containing impurities in the system will undergo chemical reactions with the catalyst, poisoning the active sites and leading to a decrease in activity [134]. The high-temperature environment will also change the crystal structure of the catalyst, causing the migration or volatilization of the active components and damaging its active structure [135]. In order to improve the stability and service life of the catalyst, the risk of deactivation can be reduced by optimizing the preparation process and precisely regulating the reaction temperature, space velocity, and other conditions. At the same time, the problem of by-products cannot be ignored. By-products such as aldehydes and ketones generated by incomplete oxidation may be more toxic, which seriously affects the catalytic effect and environmental safety [136].

From the perspective of energy efficiency and cost, improving energy efficiency and reducing costs are key challenges. To improve energy efficiency, it is necessary to conduct in-depth research on the structure of the active sites of the catalyst and the reaction mechanism, optimize the formulation to reduce the activation energy of the reaction, achieve efficient reactions at low temperatures, and reduce energy consumption. At the same time, it is also necessary to improve the reactor and optimize the mass transfer and heat transfer processes. In terms of reducing costs, inexpensive and readily available raw materials can be found to replace expensive metal elements, and efficient and low-cost preparation processes can be developed to reduce energy consumption and resource waste.

The problem of secondary pollution also restricts the application of perovskite catalysts. The insufficient ability of the catalyst to adsorb and convert intermediate products will lead to their accumulation and release, resulting in secondary pollution. If the reaction conditions are not properly controlled, the incomplete oxidation of VOCs will also produce harmful by-products. In addition, leakage caused by poor equipment sealing is also one of the sources of secondary pollution. To solve this problem, it is necessary to optimize the catalyst design, enhance its ability to convert intermediate products, precisely regulate the reaction conditions at the same time, and use online monitoring technology to monitor and adjust in real time. The sealing and maintenance management of the equipment should be strengthened to prevent leakage. Future research can focus on developing in situ monitoring technology to track the reaction process and the generation of secondary pollutants in real time and can carry out interdisciplinary research to fundamentally solve the problem of secondary pollution.

### 4.2. Prospect

In the future, efforts should be dedicated to the development of new perovskite catalysts with higher activity and stability. On the one hand, through theoretical calculations and high-throughput experimental techniques, new element-doping combinations should be screened and designed, and completely new crystal structures should be explored to enhance the adsorption and activation ability of VOCs. Meanwhile, the performance of the catalyst in resisting poisoning and sintering should be improved. For example, attempts can be made to introduce some rare-earth elements or transition metals with special electronic structures to regulate the electron cloud density of the catalyst, thereby optimizing the catalytic active sites. On the other hand, composite-structured perovskite catalysts can be developed. By combining perovskite with other materials with special properties (such as carbon nanomaterials, molecular sieves, etc.), the overall catalytic performance can be enhanced through the synergistic effect, achieving more efficient removal of different types of VOCs.

In terms of the preparation process, more environmentally friendly, simple, and precisely controllable synthesis methods should be developed to achieve precise control over the microstructure and morphology of the catalyst, thus improving the consistency and reproducibility of the catalyst performance. In the aspect of the reaction process, by integrating advanced process control technologies, real-time monitoring and intelligent regulation of parameters such as reaction temperature, pressure, and gas flow rate can be realized. Optimizing the reaction conditions can further improve energy utilization efficiency and reduce operating costs.

It should be noted that in-depth mechanism research is crucial for the development of perovskite catalysts. Using advanced characterization techniques such as in situ X-ray photoelectron spectroscopy and in situ infrared spectroscopy, the species changes, electron transfer, and the generation and transformation of reaction intermediates on the catalyst surface during the catalytic oxidation process can be tracked in real time to reveal the micro-mechanism of the catalytic reaction. On this basis, more accurate kinetic models can be established to deeply understand the influencing factors of the reaction rate, providing a solid theoretical foundation for catalyst design and process optimization. Through a deep understanding of the reaction mechanism, the current existing problems, such as reducing by-product generation and avoiding secondary pollution, can also be addressed in a targeted manner, promoting the practical application of perovskite catalysts in the field of VOC catalytic oxidation.

## 5. Conclusions

In terms of catalyst performance research, numerous experiments and theoretical analyses have shown it has excellent catalytic activity. It can promote the oxidation reaction of VOCs at relatively low temperatures, effectively reducing the reaction energy consumption. In terms of thermal stability, the inherent characteristics of the perovskite structure enable it to maintain a stable structure in a high-temperature environment and ensure the continuous exertion of catalytic performance. Moreover, when facing complex waste gas components, it exhibits strong anti-poisoning ability, greatly improving the service life of the catalyst. Meanwhile, through the doping and modification of elements at the A-site or B-site, the electronic structure and lattice oxygen activity of the catalyst can be precisely regulated, thereby achieving efficient catalysis of different VOCs. From the exploration of the reaction mechanism, with the help of advanced characterization methods such as in situ XPS and in situ infrared spectroscopy, researchers have clearly analyzed the adsorption mode of VOCs on the surface of the perovskite catalyst, which is achieved through specific interactions with the surface-active sites. Finally, with the participation of lattice oxygen, VOCs are gradually activated and ultimately oxidized into harmless substances such as carbon dioxide and water. This in-depth understanding points out the direction for subsequent catalyst optimization. In the application field, currently, perovskite catalysts have successfully achieved the efficient removal of various common VOCs such as benzene, toluene, and formaldehyde at the laboratory bench-scale stage, with considerable conversion rates. However, when moving towards large-scale industrial applications, there are still many obstacles. The cost remains high, and the high costs of raw materials and preparation processes limit its wide promotion. During long-term operation, affected by the fluctuations in working conditions, its stability needs to be further improved. In addition, how to seamlessly connect with existing industrial waste gas treatment equipment is also an urgent problem to be solved. Although there are many challenges, based on their outstanding catalytic advantages and environmental protection characteristics, perovskite catalysts have great potential in the field of VOC treatment. In the future, continuous efforts are needed in aspects such as cost reduction, stability enhancement, and adaptation to industrial equipment to promote it and enable it to play a greater role in waste gas treatment.

## Figures and Tables

**Table 1:** Comparison of perovskite catalysts in different systems for the catalytic oxidation of VOCs.

Catalyst Systems	Catalysts	Doping/Loading	VOC	Ref.
A-site doping	La_0.8_M_0.2_MnO_3_	A-site: M = Ba, Ca, Ce, Mg, Sr	Ethyl acetate	[92]
	La_1−x_Al_x_MnO_3_	A-site: Al	1,2-dichloroethane	[93]
B-site doping	La_1−x_Ce_x_Mn_1−y_Cu_y_O_3_	B-site: Cu	Toluene	[94]
	SrMn_0.8_Ce_0.2_O_3_	B-site: Ce	Acetone	[95]
A- and B-site co-doping	La_0.8_A_0.2_Mn_0.3_B_0.7_O_3_	A-site: Sr/Ce B-site: Cu/Fe Co-doping	Toluene	[96]
Non-noble metal loading	LaCoO_3_/MgO	support: MgO loading: LaCoO_3_	Toluene, methane	[97]
	CrO_x_/LaSrMnCoO	support: LaSrMnCoO_6_ loading: CrO_3_	1,2-dichloroethane	[98]
Noble metal loading	Au/3DOM LaCoO_3_	support: LaCoO_3_ loading: Au	Toluene, CO	[100]
